# A new type of ant-decapitation in the Phoridae (Insecta: Diptera)

**DOI:** 10.3897/BDJ.3.e4299

**Published:** 2015-01-02

**Authors:** Brian V. Brown, Giar-Ann Kung, Wendy Porras

**Affiliations:** † Natural History Museum of Los Angeles County, Los Angeles, United States of America

**Keywords:** Tropical, behavior, Dohrniphora, Formicidae, specialized saprophagy.

## Abstract

The genus *Dohrniphora* is a hyperdiverse group of phorid flies, a family whose species are commonly characterized as generalized scavengers. The lifestyle of most species of *Dohrniphora* is unknown, although one cosmopolitan, synanthropic species, *D. cornuta* (Bigot) fits the general scavenger mold. Here we show that flies of the *D. longirostrata* species group exhibit highly specific “headhunting” behavior in which injured *Odontomachus* ants are decapitated, the heads dragged away, and females either feed on their contents or lay an egg nearby. Since most females studied lacked eggs in their ovaries, we conclude that this bizarrely specialized feeding is necessary to provide nutrients for reproduction in these flies. Our study provides further evidence that injured ants are a common, stable resource in tropical ecosystems that support a wide array of phorid flies. Such narrowly constrained lifestyles, as exemplified by exclusively feeding on and breeding in the head contents of certain ponerine worker ants, could allow the co-existence of a huge community of saprophagous flies.

## Introduction

The Phoridae are a family of over 4,000 species of small (0.4-6.0 mm) poorly-known flies, with an incredible diversity of lifestyles. Disney (1990) referred to them as the most biologically diverse family of insects, because their larvae indulge in such wide-ranging feeding habits as herbivory, predation, scavenging, and parasitism. Among the common names of some species are vivid descriptors like "coffin flies", "bee-killing flies", and "ant-decapitating flies", the last referring to species of the genera *Apocephalus* Coquillett, *Pseudacteon* Coquillett, and some other genera of the subfamily Metopininae whose larvae feed in the heads of their ant hosts, eventually causing the head to fall off. All metopinines that decapitate their hosts do so in this same way, by larval feeding, but some might also release an enzyme to dissolve the attachment between the head and the rest of the body (Porter 1998).

Here, we describe an entirely new type of ant decapitation, one that is otherwise unknown in any insect. Instead of decapitation caused by larval feeding, as in metopinines, our observation involves the activity of the adult female phorid of a different subfamily (Phorinae). The description of this lifestyle is based on earlier observations by us (Kung and Brown 2005) on females of the *D. longirostrata* group of species of the genus *Dohrniphora* Dahl. This group, consisting of 7 species (Brown 2008, Kung and Brown 2005) occurs only in the Neotropical Region. Three of these were documented to have an association with experimentally injured workers of certain genera of ponerine ants, especially the larger-sized species of *Odontomachus* Latreille. The research described herein was to further investigate and document this extraordinary interaction.

## Materials and methods

Observations were made in three places: forest fragments on a private coffee farm in southern Minas Gerais state in Brazil, near the town of Cabo Verde (CV- 21.45°S, 46.34°W), La Cangreja National Park in Costa Rica (LC- 9.68°N, 84.39°W), and La Suerre in Costa Rica (LS- 10.13°N, 83.73°W). At these sites, we observed the interaction of female *Dohrniphora longirostrata* (Enderlein) (CV), *D. oricilla* Kung & Brown (LC), and *D. conlanorum* Kung & Brown (LS) with experimentally injured (crushed with forceps) host ants *Odontomachus chelifer* (Latreille) (CV), *O. erythrocephalus* Emery (LS), and *O. baueri* Emery (LC). Observations were made throughout the day, but flies were found to be most active at dusk. Voucher specimens of both flies and ants are deposited in the Natural History Museum of Los Angeles County (LACM), as follows: *D. conlanorum* [catalog # LACM ENT 050969], *D. longirostrata* [LACM ENT 059215], *D. oricilla* [LACM ENT 212894] *O. chelifer* [LACM ENT 038451], *O. erythrocephalus* [LACM ENT 256234], *O. baueri* [LACM ENT 305128].

## Taxon treatments

### Dohrniphora

Dahl 1898

#### Biology

All species of flies had similar behavior, documented in video clips. Approximately 10 decapitations per species have been observed. Flies arrived shortly after the ants were injured, usually arriving as in copula pairs in flight, cruising back and forth above the ants. After the pair landed, males immediately departed and females approached the injured *Odontomachus*.

Female flies spent several minutes apparently assessing the degree of incapacitation of the ants. First, a fly would rapidly tap on undergrowth leaves with its body (“drumming”) while circling about 5 cm around the ant. This drumming behavior was observed only with *D. oricilla*, but might have been overlooked in other species. Next, the fly approached the ant, darting in to touch it occasionally, still circling it. Occasionally, flies would grasp antennae or legs and rapidly pull on them (Fig. 1 at 19 seconds). If ants were too active, flies retreated and approached other, more incapacitated hosts (Fig. 2).

Eventually, flies climbed on the ant body, and began to probe with their mouthparts. Each fly concentrated on the occipital region of the ant body, using their mouthparts to probe deeply through the membrane (Fig. 3). The fly was nearly constantly in motion, probing from several angles. They made in-and-out, as well as rotational head movements, apparently while they were cutting the tissue of gut tract, nerve cord, and intersegmental membrane. On some occasions, flies preceded this stage by pulling the entire ant host out of the observation area, apparently to deal with it in a more secluded location.

**Figure 4.**
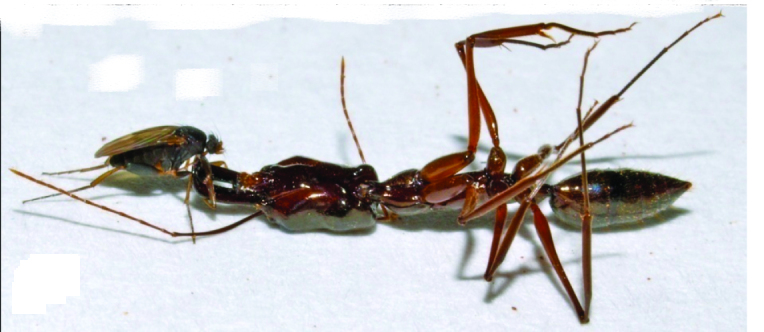
A female of *D. longirostrata* decapitating an *Odontomachus* ant near Cabo Verde, Brazil.

Eventually, the ant's head became loosened and after some tugging (Fig. 4), the fly pulled the head off the body. It then would drag the ant head as far as several meters away, by holding onto the head with its forelegs and pulling with its middle and hind legs (Fig. 4). Interactions were followed for up to 45 minutes until the fly was lost in the undergrowth. A minimum of about 8 minutes were required by the fly to decapitate an ant, although such short time periods were rarely observed. Instead, timed sessions varied widely due to interruptions in the decapitation process due to the presence of competing females, other saprophagous phorids, and other insects, especially ants. In one instance, a pair of *D. conlanorum* females were observed in a 20 s long "fight" during which they flailed each other with their forelegs while rapidly running, flying, and jumping just above the leaf litter surface.

Some flies (n=10) were captured, placed in a plastic tub with injured ants, and observed indoors. Most decapitated their hosts quickly in low light conditions, and fed upon the head capsule contents. On two occasions, flies laid a single egg 1 cm from the ant head. Injured crickets and grasshoppers (Insecta: Orthoptera) placed in the same cages were ignored by flies.

**Figure 5.**
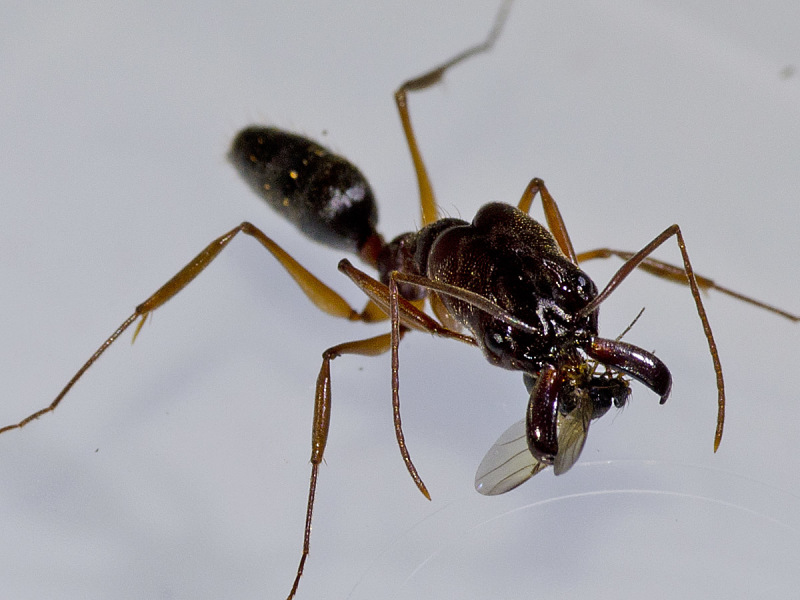
An *Odontomachus* ant eating a captured *D. conlanorum* female (photo by Inna Strazhnik).

Apparently, healthy ants are not subject to attack by *Dohrniphora* females. Under laboratory conditions, caged flies were frequently captured and crushed by ants (Fig. 5).

##### Morphology

**Figure 6.**
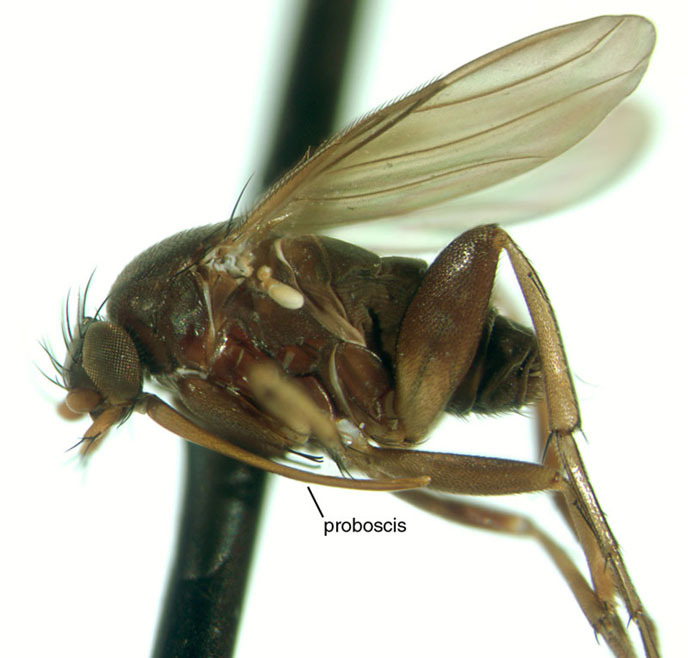
A female of *D. longirostrata*, lateral view.

**Figure 7.**
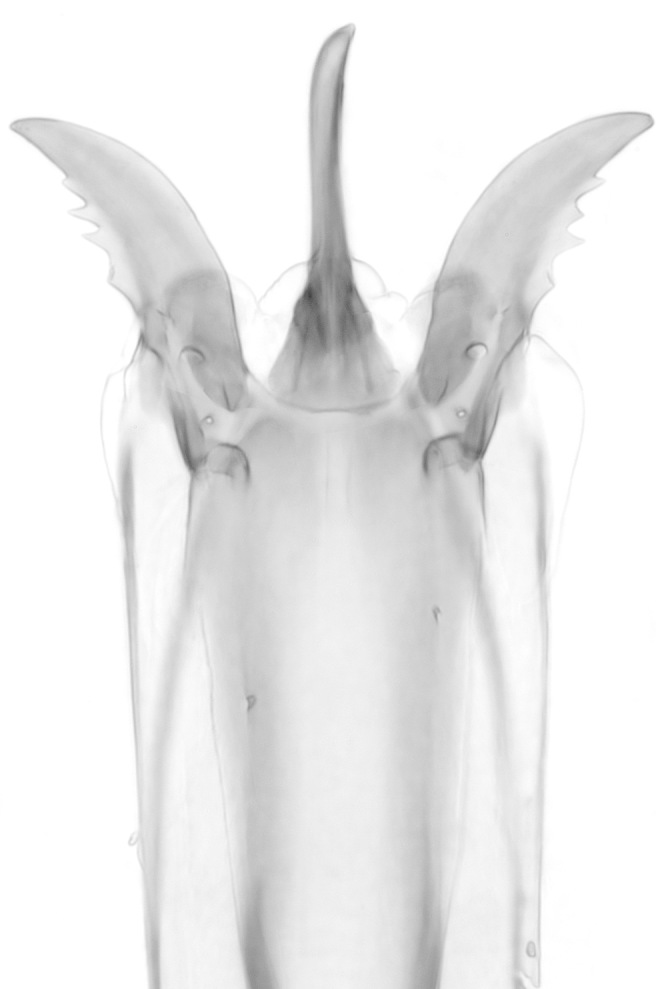
Tip of female *Dohrniphora longirostrata* proboscis.

Females of the *Dohrniphora longirostrata* species group are distinctive because of their greatly elongated proboscis, which is almost as long as their entire body (Fig. 6). In our observations, this proboscis is used to separate the ant’s head from the rest of the body. Study of the structure of the apex of the proboscis shows that the epipharynx is extremely modified as a bladed cutting organ that is used in the process of severing the ant's head (Fig. 7).

We rarely observed oviposition in our study, but it originally seemed unlikely that the flies would be engaging in this ant-decapitating behavior for any other reason than to secure food for their larvae. A single ant head appears to be the required size for the development of the single fly larva. In captivity, however, the flies were usually observed feeding on the contents of ant head capsule. More strikingly, we dissected females arriving at the injured ants (n=16) and found no mature eggs in their ovaries. As these non-gravid flies could not possibly have oviposited, we therefore conclude that female *Dohrniphora* require feeding on the contents of *Odontomachus* heads in order to mature their eggs.

## Discussion

Females of the *Dohrniphora longirostrata* group decapitate injured *Odontomachus* ants in tropical Central and South America, both for their larvae but apparently also to feed themselves and allow development of eggs. Such feeding behavior is known for other non-gravid parasitoid flies feeding on their injured host ants (Brown 2000), especially *Apocephalus* species. For *Dohrniphora* females, this behavior is laborious and time-consuming, but is apparently a viable way for them to obtain food. The species *D. longirostrata* is common in Atlantic forests in Brazil, and in other localities *D. longirostrata* group species are among the commonest *Dohrniphora* in Malaise trap samples (Brown and Kung 2007). Injured *Odontomachus* hosts must be common in the environment for flies to reach such densities. Indeed, recent work suggests that injured ants in general are a reliable source of hosts and nutrients for phorid flies (Segura and Brown 2014). Over the last 30 years, the authors have been crushing ants and other arthropods in tropical forests in eight countries of South and Central American, resulting in about 120 distinct collecting records of nearly 2500 specimens (Suppl. material 1). This vast resource of data shows that, in addition to the *D. longirostrata* species group, at least 70 species of the *Apocephalus miricauda* group feed and oviposit on injured ants (Brown 2000), as do the 10 known species of the metopinine genera *Rhyncophoromyia* Malloch, 14 *Diocophora* Borgmeier (Brown 1999), and some others (Disney and Maschwitz 2000, Brown 2010, Disney and Brown 2003). Milichiid, chloropid and ceratopogonid flies are also frequently attracted, apparently to feed. Possibly, as was postulated for parasitoids of the ant *Paraponera clavata* (Brown and Feener 1991), frequent inter-colony aggression (Segura and Brown 2014) provides the large number of injured workers necessary for these flies to breed so profusely. Regardless of the source in nature, the specificity of *D. longirostrata* group species to *Odontomachus* ants amidst the hundreds of records of phorid flies attracted to injured ants is notable. These flies were similarly never attracted to injured grasshoppers, katydids, or termites, all of which are part of our normal field protocol and attract many other phorids (including other *Dohrniphora*).

It is common in tropical forests for up to 50-100 species of *Dohrniphora* to be co-existing (Brown and Kung 2007, Brown and Kung 2010). These flies are often dismissed as scavengers, but if so, the number of sympatric congeners, plus hundreds of other species of phorid “scavengers” pursuing the identical saprophagous lifestyle is difficult to explain. Extremely specialized natural histories, like that of the *D. longirostrata* species group probably allows such a high diversity of saprophagous flies to co-exist in tropical forests.

## Supplementary Material

Supplementary material 1 Records of phorid flies attracted to injured arthropods from LACM collection Data type: association records; full data available from discoverlife.org
File: oo_35167.csv Brown, Kung, & Porras

XML Treatment for Dohrniphora

## Figures and Tables

**Figure 1.** A female of *D. oricilla* assessing injured *Odontomachus* ants at La Cangreja NP, Costa Rica.

**Figure 2.** A female of *D. conlanorum* processing and eventually decapitating an injured *Odontomachus* ant at La Suirre, Costa Rica.

**Figure 3.** A female of *D. oricilla* cutting the head off an injured *Odontomachus* ant at La Cangreja NP, Costa Rica.
